# The Dessau workshop on bioaccumulation: state of the art, challenges and regulatory implications

**DOI:** 10.1186/s12302-015-0067-0

**Published:** 2015-12-21

**Authors:** Gabriele Treu, Wiebke Drost, Ulrich Jöhncke, Caren Rauert, Christian Schlechtriem

**Affiliations:** 1 Umweltbundesamt (UBA), PO Box 1406, 06844 Dessau-Rosslau, Germany; 2 Fraunhofer Institute for Molecular Biology and Applied Ecology (IME), PO Box 12 60, 57377 Schmallenberg, Germany

**Keywords:** Regulation of chemicals, Assessment, Prediction, BCF, BMF, Test systems, Accumulation processes, 3R

## Abstract

Bioaccumulation plays a vital role in understanding the fate of a substance in the environment and is key to the regulation of chemicals in several jurisdictions. The current assessment approaches commonly use the octanol–water partition coefficient (log *K*_OW_) as an indicator for bioaccumulation and the bioconcentration factor (BCF) as a standard criterion to identify bioaccumulative substances show limitations. The log *K*_OW_ does not take into account active transport phenomena or special structural properties (e.g., amphiphilic substances or dissociating substances) and therefore additional screening criteria are required. Regulatory BCF studies are so far restricted to fish and uptake through the gills. Studies on (terrestrial) air-breathing organisms are missing. Though there are alternative tests such as the dietary exposure bioaccumulation fish test described in the recently revised OECD test guideline 305, it still remains unclear how to deal with results of alternative tests in regulatory decision-making processes. A substantial number of bioaccumulation fish tests are required in regulation. The development of improved test systems following the 3R principles, namely to replace, reduce and refine animal testing, is thus required. All these aspects stress the importance to further develop the assessment of bioaccumulation. The Dessau Workshop on Bioaccumulation which was held from June 26th to 27th 2014, in Dessau, Germany, provided a comprehensive overview of the state of the art of bioaccumulation assessment, provided insights into the problems and challenges addressed by the regulatory authorities and described new research concepts and their regulatory implications. The event was organised by UBA (Dessau, Germany) and Fraunhofer IME (Schmallenberg, Germany). About 50 participants from industry, regulatory bodies and academia listened to 14 lectures on selected topics and joined the plenary discussions.

## Background

The identification and scientific assessment of compounds that bioaccumulate in organisms and biomagnify in food webs are important aspects in the regulation of chemicals in several jurisdictions, such as Regulation (EC) No 1107/2009 [1], the Regulation on classification, labelling and packaging of substances and mixtures [2], and the Regulation concerning the registration, evaluation, authorisation and restriction of chemicals (REACH) [3]. Bioaccumulation also plays an important role in terms of evaluating secondary poisoning. To date, the vast majority of research on bioaccumulation has been conducted on aquatic organisms and uptake through the gills. Bioconcentration studies are commonly carried out according to the organisation for economic cooperation and development (OECD) test guideline (TG) 305 [4] which was recently revised and now provides an additional test for dietary uptake. Generally, a high amount of test animals is used for bioaccumulation studies. Thus, the development of improved testing strategies following the 3R principles, namely to replace, reduce and refine animal testing, [5] is required. This may involve the use of invertebrate species, in vitro approaches or in silico modelling. Recent scientific progress has yielded a large number of strategies and concepts for assessing chemical bioaccumulation. To address these issues, a workshop, organised by the German Federal Environment Agency (UBA) and Fraunhofer IME in June 2014, focused on bioaccumulation and its current developments and challenges in the regulatory context. In several talks, the results of UBA funded research projects on bioaccumulation issues were presented. Participants from academia, industry and regulatory bodies discussed which refinements of the test systems and screening approaches would be necessary or useful and which additional research is needed to improve bioaccumulation assessment (B-assessment).

## Review

### Refinement of B-assessment on aquatic organisms (OECD TG 305)

#### Lack of regulatory criteria for the dietary tests

The bioconcentration factor (BCF), as determined by OECD TG 305, is the standard endpoint in bioaccumulation assessment. Most substance regulations refer to a BCF. However, depending on the properties of the test items the performance of flow-through fish tests can be challenging. For instance, the performance of a fish test according to OECD TG 305 requires the possibility of preparing stable, measurable dissolved aqueous concentrations of the test substance. For highly hydrophobic substances (log *K*_OW_ > 5 and a solubility below ~0.01–0.1 mg/L), testing via aqueous exposure becomes increasingly difficult. In 2012, the dietary fish test has been added to the OECD TG 305 to provide a bioaccumulation test for substances that cannot be tested by aqueous exposure. For highly hydrophobic substances the dietary test is recommended, provided that the test is consistent with the relevant regulatory framework and risk assessment needs [4]. The dietary approach yields both a biomagnification factor (BMF) and an elimination rate constant for the chemical in focus. However, defined regulatory cut-off criteria for the biomagnification potential of chemical compounds are still missing and question the benefit of the BMF approach for the regulatory application. Being able to estimate a BCF from the data generated in the dietary study would meet the regulatory need and justify the use of animals. However, apart from the available depuration rate constant, the calculation of kinetic BCF also requires a corresponding uptake rate constant, which cannot be estimated in dietary bioaccumulation studies. The uptake rate constant derived in a flow-through test refers to the uptake via the gills whereas the elimination rate constant is considered to be the same in both test system, irrespectively of the exposure route (dietary or aqueous). Many suggestions and models have been developed to predict the uptake rate constant.

Brooke et al. [6] evaluated the available models for the calculation of uptake rate constant and came to the conclusion that uncertainty in the estimated uptake rate constant was relatively large, however, even for the best performing methods. This was confirmed by investigations on the dependency of BCF and BMF estimates on uptake and elimination presented by Ralph Kühne, UFZ, Leipzig. A literature search for bioaccumulation data from aqueous and dietary exposure fish tests was conducted with a special focus on uptake and elimination estimates. A validated data set for more than 300 individual chemical compounds with unique organic chemical structures was obtained. It was shown that uptake rate constants from aqueous exposure fish tests described in the literature showed a clear relationship with the respective kinetic BCF. However, if uptake rate constants were estimated based on different model predictions, none of them yielded sufficient results and consequently no dependency of estimated *k*_1_ and experimental BCF was found. The results presented by Ralph Kühne showed that the application of the model available to estimate the elimination rate constant only lead to a weak relationship with kinetic depuration rate constant. Merely a trend could be obtained from the Arnot and Gobas approach [7, 8] when biotransformation was considered. Interestingly Inoue et al. [9] found that there is high correlation (*r*_2_ = 0.873) between experientially derived BCF_L_ and BMF_L_ of nine poorly water soluble test substances, suggesting that the uptake route (by way of water or diet) might have no influence on bioconcentration and biomagnification potentials of the test substances. They concluded that elimination of the chemical may be the dominating factor, which in theory, is independent of the uptake route [10].

Concluding on the uncertainties of the aforementioned approaches it remains questionable whether calculated BCF values from data obtained in a feeding study are suitable and it seems more targeted using the elimination rate constant as alternative parameter for bioaccumulation and to establish adequate regulatory threshold for the elimination rate constant, rather than trying to recalculate bioaccumulation parameters derived in different test systems.

#### Advantages of using passive dosing in BCF tests

Under such circumstances, the estimation of BCFs also for highly hydrophobic organic chemicals (HOCs) in flow-through fish tests might remain essential in the future. A column elution method was developed by Fraunhofer IME to generate stable concentrations of HOCs for aquatic bioconcentration studies which can be maintained throughout an extended uptake period of up to 60 days. Bioconcentration studies with test items characterized by high [hexachlorobenze (HCB), *o*-terphenyl] and very high [PCB 153, dibenzo(a,h)anthracene] lipophilicity were carried out. The results of the BCF studies on rainbow trout (*Oncorhynchus mykiss*) presented by Christian Schlechtriem, Fraunhofer IME, Schmallenberg, show that aqueous exposure bioaccumulation fish tests according to OECD TG 305 can be carried out with HOCs. Organic matter in the test water can have a high impact on the bioavailability of HOCs in bioaccumulation studies. Liquid–liquid extraction (LLE) is commonly applied in bioconcentration studies and yields total analyte concentrations.

#### Advantages of using SPME in BCF tests

By the extraction of non-bioavailable HOC bound to particulate and dissolved organic matter, LLE may overestimate the bioavailable aqueous HOC concentration and thus underestimate the true uptake reducing the robustness of the fish test. In contrast, solid-phase microextraction (SPME) can be used to measure freely dissolved analyte concentrations representing the bioavailable concentrations in bioconcentration studies and help to assess the presence of non-bioavailable molecules bound to organic matter in the test system [11]. It was shown by Leonard Böhm, Justus Liebig University Giessen, that organic matter from BCF studies such as feed and detritus has a high sorption potential for HOCs [12]. Based on the results of this study and the following application of SPME during the above-mentioned BCF studies, the recommendation of SPME for measuring aqueous HOC concentrations was included in the revised OECD TG 305.

### Refinement of B-assessment on non-aquatic organisms

The vast majority of research on bioaccumulation has been conducted on aquatic organisms and uptake through the gills. Far less is known about B-assessment of (terrestrial) air-breathing organisms and about the long-term bioaccumulation in terrestrial food chains and ecosystems even though exposure of non-aquatic species to certain chemicals is very likely, e.g., by direct application (pesticides) or indirectly via application of contaminated sewage sludge on agricultural sites. Up to now, the only established tests for B-assessment on air-breathing animals are conducted with terrestrial oligochaetes (OECD 317, [13]). One shortcoming is that the test does not distinguish between bioconcentration (uptake of pore water via skin) and dietary bioaccumulation (uptake via food). It has been suggested that currently there are limited data to support setting definitive criteria for terrestrial bioaccumulation. Hence, there is a broad agreement that new comprehensive testing and assessment strategies are needed for non-aquatic organisms in order to consider environmental compartments other than aquatic which are also exposed to chemicals. Due to the high octanol–air partition coefficient (*K*_OA_) and corresponding low rate of respiratory elimination to air of some compounds it was suggested to integrate new screening criteria (log *K*_OA_ > 5 and log *K*_OW_ > 2) in the PBT screening within the different EU regulatory frameworks. Previous studies [14] have shown that substances with a low log *K*_OW_ may biomagnify to a high degree in food webs containing air-breathing animals (including humans).

Based on the assumption that depuration is independent of the uptake pathway, Kai-Uwe Goss, UFZ, Leipzig suggested the elimination half-life (EL0.5) as an alternative metric for bioaccumulation which could be applied to air- and water-breathing animals. The elimination rate constant is valuable for all uptake routes and would harmonise all bioaccumulation metrics [15]. Additionally EL0.5 is equivalent to the depuration rate constant *k*_2_ that is measured in various bioaccumulation and bioconcentration tests already. Accordingly, Brooke and Crooke suggested to using the depuration rate constant as alternative bioaccumulation metric and proposed that a *k*_2_ of 0.178 day^−1^ would reflect a BCF of ≥2000 L/kg and *k*_2_ of 0.085 day^−1^ a BCF of ≥5000 L/kg [16]. This approach for identifying substances as B or vB based on the depuration rate constant appears to show promise, with a large proportion of the available data set being correctly categorised for the training and validation data sets analysed [16]. Knowledge of physicochemical properties combined with an understanding of physiological processes are important factors for reasonable estimates of elimination. Albeit, additional confounding factors such as individual feeding behaviours and food preferences not related to physiology or the physicochemical properties of the compounds would make uptake kinetics less predictable. Based on a feeding rate of 1 % of the organism’s weight per day, assuming the same fugacity capacity of the organism and its diet for the studied chemical and an uptake efficiency from food of 100 %, an EL 0.5 of 70 days as a trigger value for high bioaccumulation was suggested (adjustments for lower efficiencies can be made, [15]). This would guarantee a BMF < 1 in terrestrial and aquatic food chains for a reasonable, mostly worst-case uptake scenario. It was noted in the plenary discussion that several issues are still up for debate: such as surface-volume ratio of organisms or varying metabolic capacity of different species. Generally, a one-compartment kinetic for all elimination processes is assumed. In the case of slower and two compartment elimination, this would have to be taken into account.

Furthermore, the participants agreed that allometrical scaling would be needed to adjust size and mass differences of individuals or species and to improve the comparability of the results.

### Reduction of animals in bioaccumulation studies

A substantial number of bioaccumulation fish tests are conducted regularly. To reduce the need for animal testing, the prediction of bioaccumulation by in vitro approaches determining chemical metabolism has been considered. Due to resource limitations associated with running a full OECD TG 305, for most chemicals bioaccumulation assessment is already performed by using log *K*_OW_-based (Q)SARs or other models. Since in silico BCF models often neglect the contribution of fish metabolism as a clearance mechanism, they might overestimate the bioaccumulative potential of a chemical and, as a consequence, trigger unnecessary in vivo tests. Inclusion of biotransformation rates would enhance the reliability of the in silico models for BCF prediction. Marlies Halder, EC DG JRC, Ispra, presented a summary on current investigations in this field including an in vitro approach using rainbow trout S9 liver fractions [17, 18] or rainbow trout hepatocytes [19]. Accumulation of a chemical in an organism is the result of several physiological processes, namely absorption, distribution, metabolism, and excretion (ADME). The in vitro approaches aim at determining loss of substance via metabolism, and use a model to extrapolate from in vitro to whole body transformation rates [19]. This is useful information which adds to the overall picture of bioaccumulation. Some regulations, e.g., REACH [3], expressively consider all available data (weight of evidence approach). In vitro test on metabolic rates can be used to predict “baseline” BCF and thus seem to be very useful for screening chemicals for bioaccumulative properties. However, so far the in vitro approaches may only be used as supportive data in assessment given the limited experience with these in vitro systems at present. In order to facilitate broad regulatory use of these in vitro methods, standardised protocols and evidence of their validity are needed. Arnot et al. [20] made a first step by developing and applying methods to provide guidance in the selection of whole-body in vivo metabolic biotransformation rate constants and to explore some factors that may contribute to observed differences in metabolic rates, such as chemical structure and fish species. A multi-laboratory ring trial coordinated by the ILSI-HESI Bioaccumulation Committee is currently carried out to assess the reliability, transferability, and predictive value of two in vitro systems using either rainbow trout S9 liver fractions or cryopreserved rainbow trout hepatocytes as in vitro systems.

Reduction of animals in bioaccumulation studies may be also achieved by the co-use of sample material collected in other studies carried out for regulatory purposes. For instance, “Early life stages” fish tests (OECD TG 210, [21]) and chronic fish tests (OECD TG 212, [22]) are carried out to define the lethal and sub-lethal effects of chemicals on the early life stages of the species tested. In the plenary discussion, the use of both tests was suggested to determine a “tentative steady state BCF” by analysing the body burden in the test animals at the end of the exposure period, instead of killing the fish without further use. This tentative BCF could be used as a possible trigger either for a full BCF test or as a justification for waiving the full test, if testing conditions in the toxicity test were adequate for BCF testing (e.g., non-toxic concentration, low TOC as specified in the OECD TG 305). One suggested use was for substances with a log *K*_OW_ between 3 and 4.5, where a BCF test would not be mandatory in all substance regulations. In theory, this approach may help to reduce vertebrate tests by using one test in two ways.

An approach presented by Christoph Schäfers, Fraunhofer IME, Schmallenberg, aims to replace fish as test organisms in bioaccumulation studies by a non-vertebrate species, the freshwater amphipod *Hyalella azteca.* Investigations were carried out to evaluate the potential of this epi-benthic amphipod to be used as alternative test organism for bioaccumulation studies, providing the opportunity to explain bioaccumulation from water (bioconcentration). The uptake and accumulation of several lipophilic substances from water were investigated. Animals collected during the bioaccumulation studies were analysed for their tissue concentrations. Based on the kinetic study design the depuration and uptake rates for the test items were determined which were further used to calculate species-specific BCF estimates. The results were compared with BCF values obtained from fish bioaccumulation studies in rainbow trout (*O. mykiss*) which were previously carried out according to the revised OECD TG 305. Additional animals were collected during the studies for lipid determination. Tissue concentrations were normalised to a lipid content of 5 % to make the results obtained from bioaccumulation tests using fish or *Hyalella* comparable. The results show that bioconcentration factors determined in *Hyalella* bioaccumulation studies are similar to those obtained from fish tests. Steady state tissue concentrations were reached in all studies within significantly shorter uptake periods compared to fish studies on the same test items. The collection of only ten adult amphipods resulted in pooled biomass sufficient to quantify tissue concentrations. Uptake and elimination rates could be determined for *Hyalella* used in bioconcentration testing. Compared to studies on rainbow trout significantly shorter depuration periods were required to reach 90 % elimination of accumulated test items. Further investigations are required to elucidate the metabolism of *Hyalella azteca*. The results obtained so far show that bioaccumulation studies with *H. azteca* may support animal welfare considerations using a non-vertebrate species, improve efficiency and reduce costs for BCF testing.

### Replacement of bioaccumulation studies

Partitioning between octanol as a hydrophobic and water as a hydrophilic phase (log *K*_OW_) is currently used as an indicator for bioaccumulation in regulation, based on the general assumption that the lipid content in the organism, or its tissue, has a sorptive capacity comparable to that of solvent octanol and is the dominant accumulation phase. Physiologically based toxicokinetic (PBTK) models can be used to compare measured bioconcentration parameters against bioconcentration parameters predicted based on lipid distribution. In a study on rainbow trout presented by Thomas Preuss, RWTH Aachen, a good prediction of measured whole body bioconcentration was found for moderately lipophilic chemicals with the model assuming lipid triggered distribution. Interestingly, model predictions for perfluorinated alkylated substances (PFASs) were not markedly different compared to the organ distribution or bioconcentration of chemicals for which lipid triggered distribution is assumed. In conclusion, whole body bioconcentration for 75 % of all compounds (including PFASs) could be explained assuming lipid triggered distribution. The study showed that the analysis of bioconcentration and bioaccumulation data with PBTK models could improve the understanding of the underlying mechanisms and reduce uncertainty of regulatory decision making in the future.

However, using log *K*_ow_ as an indicator for bioaccumulation may be too simplistic, considering the diverse structures of environmental chemicals and the complexity of biological matrices. In the past years, the sorptive capacities of major biological phases—storage lipid, membrane phospholipids, and proteins—for neutral organic chemicals have been systematically investigated, (e.g., [23]). Chemicals considered include a range of nonpolar and polar chemicals covering a large structural diversity. The polyparameter linear free energy relationship (PP-LFER) models were used to evaluate and predict respective lipid–water and protein–water partitioning coefficients. As summarised in the presentation of Satoshi Endo, UFZ, Leipzig, significant differences in accumulation properties between membrane and storage lipids [24, 25] exist. Combining both fractions as “the total lipid” can lead to inaccurate predictions and interpretations of field bioaccumulation. The comparison between lipids and proteins suggests that for polar compounds, not only lipids but also proteins can be the significant sorbing phase in lean tissues such as muscle [26].

Though the lipid–octanol model is not mechanistically valid for all kind of chemicals, it appears to provide at least an order of magnitude estimate in most cases. The PP-LFER is however a more differentiated tool to predict partitioning into different phases. It can improve the prediction of lipid- or protein-based bioaccumulation and can provide valuable insights into the roles of lipids and proteins in bioaccumulation and the possibility of tissue-specific accumulation of particular chemicals. While the sorption of neutral chemicals correlates well with log *K*_OW_, the sorption of ions is more complex and not well understood so far—despite the fact that 49 % of the preregistered industrial chemicals under REACH [27] are charged compounds and may accumulate significantly in membranes. So far there is no approach to estimate the bioaccumulation potential of hydrophobic ions in regulation. For dissociating substances, log *D* can be used to assess the portion of the neutral and ionic forms of the substance and its partitioning between water and octanol depending on the pH in the environment. Hydrophobic ions may accumulate significantly in membranes and are potentially bioaccumulative due to their strong sorption to membrane lipids. This partitioning cannot be reliably modelled using bulk solvents. Fundamental differences between bulk-phase and membrane-water partitioning make a mechanistic modelling approach necessary for the treatment of ions. The use of the model COSMOmic for the prediction of partitioning of ionic species in membranes [28] in combination with an optimised membrane dipole potential was recommended as an alternative screening tool by Kai Bittermann, UFZ, Leipzig.

Non-lipid based processes may significantly (>1 log unit) increase the bioaccumulation of chemicals beyond the extent to be expected from their log *K*_OW_. However, current screening criteria likely miss such compounds and their risks may not be recognised. Which additional processes (beyond log *K*_OW_) contribute to the bioaccumulation of which classes of chemicals and how they can be recognized was the central question of a project presented by Monika Nendza, AL-Luhnstedt. An exploratory data analysis was carried out with BCF data (OSIRIS PROJECT 2007–2011, http://www.ufz.de/osiris) relative to the established *K*_OW_-based QSAR model by Veith et al. [29]. Substances like PFAS are suggested to bioaccumulate by protein binding. However, the results of the data analysis show that protein binding does not correlate with increased bioaccumulation beyond log *K*_OW_. Protein binding is dominated by log *K*_OW_ and correlates with log BCF. Accumulation may be due to protein binding, but the protein binding can be predicted from log *K*_OW_. From an overall bioaccumulation perspective, protein binding does not provide additional information about the quantitative bioaccumulation of substances beyond log *K*_OW_. However, information on the partitioning of substances as described above can greatly improve the understanding of the mechanisms leading to bioaccumulation.

The bioaccumulation of surfactants in fish shows no correlation with log *K*_OW_ and is often lower than the log *K*_OW_-based estimates. For the most part, the distribution and accumulation of surfactants in organisms depends on their absorption at biological interfaces and is affected by metabolism and excretion. However, the surface activity may cause absorption to food items and can contribute to an increased uptake by diet. As yet, quantitative information on the dietary uptake of surfactants is too limited for general conclusions.

An increased or decreased bioaccumulation may also be explained by membrane specific absorption processes. To assess which different transporter systems may contribute to enhanced or decreased uptake, the outcome of a literature search on information of general membrane transport properties was presented by Ariane Zwintscher, Fraunhofer ITEM, Hannover. Especially the transport via carrier (secondary active transport) might lead to an increased uptake, which does not correlate with the lipophilicity of the substances. Then, an underestimation of BCF from log *K*_OW_ may result. To predict the uptake potential of substances, several in vitro and in silico models have been developed over the last decades (e.g., PAMPA assay, Caco-2 assay, Gill cell culture, etc.). The human intestinal Caco-2 cell line has been extensively used over the last 20 years as a model of the intestinal barrier. As the Caco-2 cell assay comprises both active and passive transport properties it is a promising in vitro assay to investigate uptake and efflux processes. To explore the relationships between bioaccumulation and uptake in the gastro intestinal tract (GIT), experimental apparent permeability coefficients (*P*_app_) values obtained from Caco-2 cells were analysed and presented by Ralph Kühne, UFZ, Leipzig. Screening criteria based on the modelled apparent uptake in the Caco-2 assay were suggested as useful indicators of increased bioaccumulation potential. A workflow to integrate the different screening criteria in the bioaccumulation assessment under REACH was presented. Nevertheless, since the Caco-2 assay is a human intestinal cell line, the transferability of Caco-2 results to bioconcentration in fish and other aquatic organisms remains to be verified, even though current literature indicates that the gills contain many of the enzymes involved in xenobiotic metabolism and transport proteins, e.g., [30, 31].

## Conclusions

The Dessau Workshop on bioaccumulation gave a comprehensive overview of the state of the art of B-assessment, provided insights into the problems and challenges faced by the regulatory authorities and described new research concepts and their regulatory implications. In brief, the use of the standard BCF test following OECD TG 305 has been demonstrated to generate reliable results with aquatic exposure for the majority of neutral, lipophilic organic substances. In addition, using column generated concentrations and keeping the total organic carbon in the test system as low as possible, flow-through tests can be also carried out with highly lipophilic test items (HOCs)SPME measurements used for the extraction of freely dissolved concentrations can provide important insights into the presence of molecules bound to organic matter and thus the bioavailability of HOCs in flow-through tests. Consequently the recommendation of SPME for measuring aqueous HOC concentrations has been included in the revised OECD TG 305. The elimination half-life (EL0.5) might be considered as a potential metric which could harmonise the different bioaccumulation metrics. The prediction of bioaccumulation by in vitro approaches such as fish S9 liver fractions and primary hepatocytes shows high potential to reduce the need for animal testing. Alternatively, bioconcentration studies with the freshwater invertebrate *H. azteca* may support animal welfare considerations using a non-vertebrate species. Physiologically based toxicokinetic (PBTK) models can improve the understanding of the underlying mechanisms bioaccumulation processes and reduce uncertainty of regulatory decision in the future. Organism–water and tissue–water partitioning coefficients for many neutral chemicals can be accurately predicted by combination of compositional information and PP-LFER models. In contrast, sorption of ions is complex and still not well understood. The use of the model COSMOmic in combination with optimised membrane dipole potentials is recommended for charged compounds. The use of new screening criteria for non-lipid accumulating substances may help to identify such compounds which pose a risk for bioaccumulation. The acquisition of information on bioaccumulation/biomagnification potential and the mechanisms involved as well as the potential to reduce the need for vertebrate testing should be explored further at all levels, and incorporated into a comprehensive testing and assessment strategy that goes beyond a mere bioconcentration factor. Concluding, we strongly recommend that the proposed amendments will be considered for implementation into the current and future guidance documents.

## Abbreviations

BAF: bioaccumulation factor
BCF: bioconcentration factor with aqueous exposure
BCF_W_: wet-weight based BCF
BCF_L_: lipid-based BCF
BMF: dietary bioaccumulation factor
DF: distribution factor
GLP: good laboratory practice
GIT: gastro intestinal tract
HOC: hydrophobic organic compound
(Q)SAR: (quantitative) structure-activity relationship
LLE: liquid–liquid extraction
*K*_OA_: octanol–air partition coefficient
*K*_OW_: octanol–water partitioning coefficient
OECD: organization for economic co-operation and development
PBTK: physiological based toxicokinetic
PCB: polychlorinated biphenyls
REACH: regulation on registration, evaluation, authorization and restriction of chemicals
SPME: solid-phase microextraction
TG: technical guideline
